# FOSTER—An R package for forest structure extrapolation

**DOI:** 10.1371/journal.pone.0244846

**Published:** 2021-01-28

**Authors:** Martin Queinnec, Piotr Tompalski, Douglas K. Bolton, Nicholas C. Coops

**Affiliations:** 1 Faculty of Forestry, University of British Columbia, Vancouver, British Columbia, Canada; 2 Department of Earth & Environment, Boston University, Boston, Massachusetts, United States of America; Indiana State University, UNITED STATES

## Abstract

The uptake of technologies such as airborne laser scanning (ALS) and more recently digital aerial photogrammetry (DAP) enable the characterization of 3-dimensional (3D) forest structure. These forest structural attributes are widely applied in the development of modern enhanced forest inventories. As an alternative to extensive ALS or DAP based forest inventories, regional forest attribute maps can be built from relationships between ALS or DAP and wall-to-wall satellite data products. To date, a number of different approaches exist, with varying code implementations using different programming environments and tailored to specific needs. With the motivation for open, simple and modern software, we present FOSTER (Forest Structure Extrapolation in R), a versatile and computationally efficient framework for modeling and imputation of 3D forest attributes. FOSTER derives spectral trends in remote sensing time series, implements a structurally guided sampling approach to sample these often spatially auto correlated datasets, to then allow a modelling approach (currently k-NN imputation) to extrapolate these 3D forest structure measures. The k-NN imputation approach that FOSTER implements has a number of benefits over conventional regression based approaches including lower bias and reduced over fitting. This paper provides an overview of the general framework followed by a demonstration of the performance and outputs of FOSTER. Two ALS-derived variables, the 95^th^ percentile of first returns height (*elev_p95*) and canopy cover above mean height (*cover*), were imputed over a research forest in British Columbia, Canada with relative RMSE of 18.5% and 11.4% and relative bias of -0.6% and 1.4% respectively. The processing sequence developed within FOSTER represents an innovative and versatile framework that should be useful to researchers and managers alike looking to make forest management decisions over entire forest estates.

## 1 Introduction

Up-to-date and extensive assessment of forest structure and resources are crucial to support sustainable forest management practices and wall-to-wall monitoring programs [1,2]. The past decade has seen a revolution in the availability of 3-dimensional (3D) information on forest structure through the increased application of light detection and ranging (lidar), also known as airborne laser scanning (ALS), which provides a 3D point cloud derived from laser returns reflected back from the forest structure [3]. Even more recently, digital aerial photogrammetry (DAP)-based approaches reconstructing vegetation structure from overlapping images to generate a product analogous to an ALS point cloud are being incorporated into forest inventory frameworks [4]. Independent variables derived from these point clouds can be used to characterize forest stand structure and can be used to build relationships with several inventory attributes [5–7]. However, despite these recent developments, forest inventories undertaken using ALS or DAP can be challenging to undertake, especially over large areas, such as in Canada, which has 347 million ha [8] of forest that require some degree of monitoring and assessment. The uneven uptake of the technology has resulted in targeted acquisitions usually supported by private companies for managed forest units, which are not systematic (with a number of exceptions [9]) resulting in a patchwork of data availability, data acquisition parameters and acquisition dates. Extensive transects of ALS data [10] and spaceborne lidar missions, such as ICESat-2 [11] and GEDI [12] are being used to extend lidar data coverage beyond the targeted managed forest units. However, these broad scale lidar acquisitions provide samples of forest structure across a range of forested ecosystems rather than a wall-to-wall mapping.

To address the need for wall-to-wall estimates, imagery acquired from spaceborne platforms can be used as the base upon which to extrapolate 3D forest attributes. Recent advances in pre-processing workflows such as cloud masking [13] and surface reflectance retrieval [14] coupled with increased computing power capable of processing large volumes of data have enabled the generation of global datasets with long historical records [15]. The Landsat series of satellites, which has provided optical multispectral imagery from 1972 onwards and other satellite programs, such as Sentinel-2 from the European Space Agency, offer a wide range of data products that can be used for imputation. For decades, attributes such as stand height, merchantable volume, diameter at breast height (DBH), and basal area (BA), were imputed directly from field plots to satellite imagery [2,16,17]. Introducing these same attributes predicted from ALS data provides a conceptually simple translation to these conventional approaches and has been successfully undertaken using a number of statistical and imputation approaches.

Existing research provides a number of modeling approaches to extrapolate ALS-derived 3D forest representations using wall-to-wall satellite data. Random forest regression models, for example, were used by Wilkes et al. [18] to estimate wall-to-wall maps of canopy height using multiple satellite datasets and other climate and topographic data. Linear regression models were also used to predict unmanned aerial vehicles (UAV) DAP-derived volume from Sentinel-2 data in a Norwegian boreal forest [19]. Beside regression, nearest neighbor (NN) imputation is another approach commonly used to extrapolate forest attributes. Observations from a reference set, for which both response variables and predictor variables are available, are compared and matched to target units for which only predictor variables are available. For each target unit, its k-nearest observations (k-NN) from the reference set are determined by calculating a proximity measure based on the predictor variables. Predictions for the target units are then determined either by directly substituting the response variables values of the nearest reference observation (when k = 1), or by averaging the response variables values of the k-nearest reference observations (when k > 1). Andersen et al. [20] incorporated ALS data with Landsat imagery and synthetic aperture radar (SAR) data as independent variables to improve estimates of biomass across Alaskan boreal forest landscapes with k-NN imputation. Zald et al. [21] and Matasci et al. [22] combined ALS-derived information with Landsat pixel-based composites to produce annual forest structure estimates over all forested regions in Canada using a k-NN model. Matasci et al. [22] found that deriving forest structural attributes on an annual basis through the integration of Landsat time series and ALS data allowed for a temporal assessment of forest dynamics in both disturbed and undisturbed forest stands, by capitalizing on the detailed structural information provided by ALS data and the long-term data record of Landsat. Bolton et al. [23] imputed ALS-derived estimates of height, basal area and volume for forest in British Columbia, Canada and found that models accuracy improved with the inclusion of Landsat imagery time series metrics when compared to single-year images. Similarly, over a range of various forest types distributed across Canada, Bolton et al. [24] found that the accuracy of extrapolated forest attributes improved as the time series length (1 to 33 years) considered to calculate Landsat time series metrics increased and tended to plateau for time series length > 15 years.

The approaches above use a variety of forest structural metrics, a range of satellite data from a range of sensors, including time series data, and a variety of statistical methods for forest attributes extrapolation including k-NN imputation, random forest, and ordinary least squares regression. Moreover, it is crucial to ensure that the sample of data used for model development is representative of the entire range of forest structure occurring across the area where the model is applied. Structural guided sampling can be carried out in different ways and a standardized approach is necessary. With the motivation for open, simple and modern software, we present FOSTER (Forest Structure Extrapolation in R), an innovative, versatile and computationally efficient framework for modeling and imputation of 3D forest attributes over large areas available as an R package. FOSTER provides three key innovations beyond what is currently available to the community in the area: (1) a robust sampling method to ensure that models are developed using the full range of response variables; (2) the calculation of spectral trends summary metrics, allowing the integration of temporal change information into the imputation and (3) a state of the art implementation of a full and flexible framework where k-NN imputation can be performed within a single environment based on the method established by Bolton et al. [23].

This paper describes the general structure of FOSTER and its imputation framework. We also demonstrate how to use the package with an example illustrating both model outputs as well as FOSTER performance using a range of computing resources and data types.

## 2 Methods

The main processing tasks implemented in FOSTER aim to facilitate the identification of relationships between spectral information captured in optical data, or any other environmental information, such as terrain elevation, with the forest attributes derived using 3D point cloud data and the subsequent imputation over the broader landscape. Imputation is performed using a k-NN model, a multivariate and non-parametric approach and rigorous validation approaches are implemented to report on model accuracy. FOSTER is implemented in R, an open source, freely available and multi-platform scripting and statistical framework [25]. The package is hosted on the Comprehensive R Archive Network (CRAN).

### 2.1 Processing approach with FOSTER

**Fig 1:**
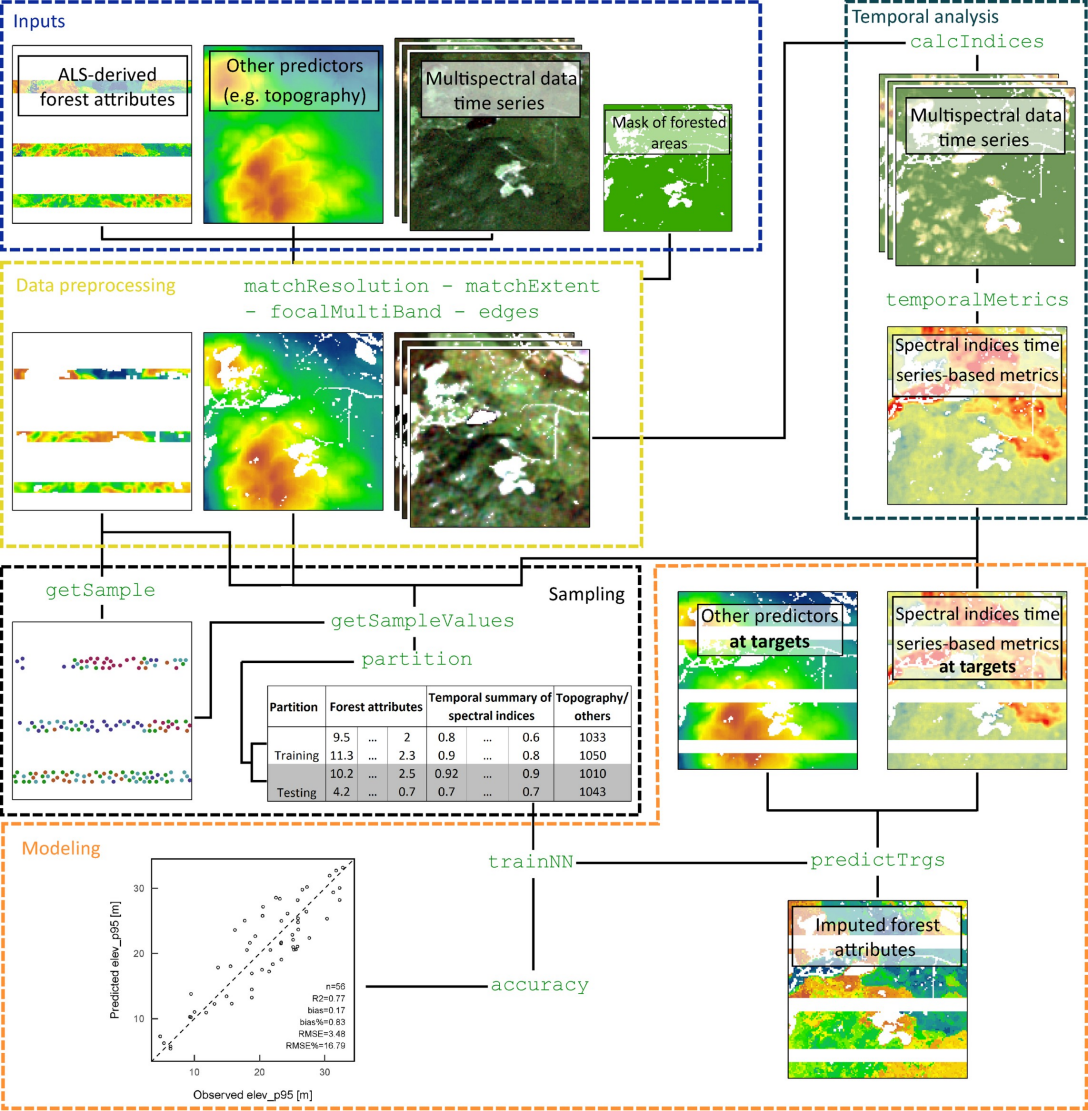
Framework around which FOSTER is designed. Each colored box represent a processing step in the workflow and the names of the functions are displayed in green.

The package is organized around functions for data preprocessing, stratified random sample selection, spectral index calculation, time series summary metrics calculation, k-NN predictive model development and their accuracy assessment, and finally response variable (i.e. forest attributes) imputation. Fig 1 shows a diagram explaining the general processing flow a user might encounter when imputing forest attributes from selected predictors that may include time series of multispectral satellite images and topographic variables. In this example, it is assumed that the response variables have already been calculated, using for example the lidR package implemented in R [26]. Methods like the area based approach [6] can be used to produce forest attributes maps but is out of the processing scope of FOSTER. Table 1 and the following subsections provide a description of the functions implemented in the package.

**Table 1:** General description of the main functions implemented in FOSTER.

	Function name	Description
Data preprocessing	matchResolution	Successively project and resample a raster layer coordinate system and spatial resolution to match a reference using bilinear of nearest neighbor methods
	matchExtent	Match the extent of a reference raster and eventually mask cells of the references that have a specific value
	focalMultiBand	Apply a function in a moving window on each band of the input raster (e.g. for smoothing)
	edges	Create a buffer (i.e. assign cells to NA values) around cells having NA values in the input raster (e.g. can be used to remove cells located on boundaries)
Multispectral data and temporal summary	calcIndices	Calculate a series of spectral indices from multispectral data
	temporalMetrics	Calculate a set of pre-defined or user defined temporal summary metrics of an annual time series of variables
Sampling	getSample	Perform a stratified random sampling of the response variables. More specifically, response variables are clustered using k-means algorithm and sample points are selected within the clusters.
	getSampleValues	Get the value of a raster cells at sample points
	partition	Split a sample into training and testing sets
Modeling	trainNN	Train a k-NN model from response and predictor variables of the training set
	accuracy	Assess the accuracy of the trained k-NN model using held-out testing set
	varImp	Return the variable importance of each predictor when random forest based k-NN imputation is used
	predictTrgs	Impute the response variables at targets

#### 2.1.1 Data preprocessing

Cell-level forest attributes, satellite imagery, and other datasets used to calculate predictors, can come from different sources and therefore have different extents and resolutions. In order to integrate these datasets together, preprocessing steps are required. Common tasks that users can encounter are resampling, cropping or projecting different raster layers in order to match a given spatial resolution and extent. In addition, masking and focal operations (also known as spatial convolution filters) can be applied to constrain modeling to specific cells or to smooth data prior to model development. The raster package [27] in R implements all the functions necessary for these tasks. However, in order to provide a *“ready-to-use”* solution, simple wrapper functions of the raster package are implemented in FOSTER.

Given a reference raster layer, the function matchResolution successively projects and resamples a raster layer in order to match the coordinate system and spatial resolution of the reference data. The function matchExtent can be used in order to match the extent of a raster to a reference and eventually mask cells that should not be considered. For example, the user might want to crop wall-to-wall satellite imagery to the study area extent and keep only cells where a forest attribute is available (e.g. masking cells from forest attribute maps with NA values).

Strunk et al. [28] found improved accuracy when linking ALS-derived forest attributes with Landsat data at a 90 m x 90 m spatial resolution compared to 30 m x 30 m. If spatial filters are desired to be applied prior to modeling, the function focalMultiBand is implemented in FOSTER and applies a filter or a function in the neighborhood of each cell and every band of the image. It is a wrapper of the focal function of the raster package and supports multiband rasters.

Based on the same principle, the function edges creates a buffer (i.e. assigns cells to NA values) around each cell with an NA value. The edges function can be used, for example, on a mask layer representing forested areas to remove all cells adjacent to forested boundaries and therefore reduce the edge effects when sampling.

The functions for data preprocessing implemented in FOSTER are all designed to perform specific tasks and group processing steps that are usually applied successively and were included in the package even though the raster package [27] and Geographic Information System or image processing software can be used to meet the users specific needs. The functions presented in the following sections form the core of FOSTER.

#### 2.1.2 Stratified random sampling

In two-stage modelling approaches where the set of reference observations is available as a gridded product (like gridded ALS metrics or forest attribute variables), it is necessary to reduce the number of reference observations for computational efficiency [29]. Moreover, due to the spatially biased nature of patchwork lidar coverages, developing any type of relationships between Landsat time series or other imagery and lidar will be biased as the lidar coverages themselves are unlikely to cover the entire forest structural conditions over the larger area. As a result, it is crucial to sample the existing lidar-derived reference datasets to build the models in an appropriate way. FOSTER utilises a non-parametric clustering approach on the available layers to develop a stratified sampling approach (recognised as state of the art in sampling frameworks for lidar data) and then sample pixels within those clusters to select a sample that is representative of all reference observations while minimizing spatial autocorrelation and avoid inflating the prediction bias. Should users wish to substitute a regression based modelling framework in FOSTER rather than k-NN, this issue becomes even more critical as regression and other statistical approaches assume independent and representative sample of the population.

In FOSTER, sampling is implemented in the getSample function that performs a k-means clustering of the normalized forest attributes and then draws random samples from the clustered observations. The k-means clustering process is based on the function unsuperClass implemented in the RSToolbox package [30] which allows for the adjustment of the clustering parameters. The number of sample points drawn from each cluster is proportional to the presence of those clusters across the reference area. Since spatial autocorrelation in the sampled points could lead to biased estimations, a distance threshold can be used in the sampling design to avoid sampling points too close to each other. The selection of an appropriate minimum value between sampled points depends on the spatial distribution of forest structural types and on the extent and pattern of the response variables. A possible approach to guide the selection of an appropriate minimum distance could be to use variograms to determine the distance at which the spatial autocorrelation of each response variable becomes negligible [31]. This distance could in turn be used as the minimum distance between sampled points in the getSample function.

The sample points are returned in a SpatialPointsDataFrame, a point features object, where each point is the centroid of the selected cells. The values of a raster layer or raster stack at the sample points can be extracted to a SpatialPointsDataFrame object using the getSampleValues function.

#### 2.1.3 Calculating spectral indices and time series-based metrics

Many predictors used in forest structure imputation studies are derived from multispectral data. Tasseled Cap indices, Normalized Difference Vegetation Index (NDVI), Normalized Burn Ratio (NBR) are some of the spectral indices commonly used [21–23,32,33]. The function calcIndices is a wrapper around the functions spectralIndices and tasseledCap implemented in the RStoolbox package [30] which supports a large number of spectral indices. calcIndices supports both raster and point layers, which is useful during model training when predictors only need to be calculated at sample point locations.

Spectral indices of vegetation usually follow seasonal patterns that repeat on an annual cycle. The trajectory of a spectral index (or any other annual time series-based predictor) over time can thus be examined from annual time series [34,35]. With the opening of the Landsat archive, algorithms have been developed to detect changes in temporal trajectories of spectral indices and relating them to forest disturbances and structural changes [35–37]. Such approaches implement complex algorithms and perform computationally demanding tasks. However, incorporating simple temporal summary metrics in forest attribute imputation consistently improves model accuracy over single year spectral indices [23,24]. The latter approach is used in FOSTER with the function temporalMetrics that calculates a set of pre-defined (median, interquartile range and Theil-Sen slope as used by [24]) or user defined metrics based on an annual time series of variables. Similarly to calcIndices, temporalMetrics supports both raster and point layers. The two functions can be executed in parallel threads to speed up computations.

#### 2.1.4 k-NN modeling and imputation

Predictor and response variables extracted at the sample locations are used to train a k-NN model, the modeling approach currently implemented in FOSTER. The package yaImpute is specifically designed for imputation problems, and modeling in FOSTER relies on its core functions yai, newtargets and impute [38]. The k-NN model calculates a proximity measure between a target and the reference observations, based on the values of the predictor variables. The measure of nearness used to calculate the proximity between observations can be determined with different methods. Among them, the random forest proximity matrix has been used in various studies for forest attributes imputation [21,23,32,39–42]. Random forest is an algorithm that can be used for both classification and regression [43,44]. A number of classification or regression trees are grown from bootstrap samples of the data. At each node, the best split among a subset of the predictor variables is used until only a defined number of observations are left in the final node. When predicting new data, the new observation is run through all the trees and a majority vote is performed to decide the value of the prediction. With imputation, we are not interested in the actual random forest prediction but in the proximity between a target and reference observations. Therefore, by calculating the proportions of trees where a new target observation falls into the same final node as a reference observation, we can define a non-Euclidean proximity measure that will be used to assign the k nearest neighbors of a target [38]. Other proximity methods like Euclidean or mahalanobis distance are also supported. Once the proximity between target and reference observation has been determined, all the response variables are imputed simultaneously to each target, based on the number of nearest neighbor *k*. If *k = 1*, the model assigns to a target observation the response variables values of its nearest reference, while if *k > 1*, the target observation gets the mean or distance-weighted mean of the response variables among the k nearest references.

The function trainNN trains a random forest-based k-NN model from the sample extracted from reference observations. The function requires the response and predictor variables for the selected sample locations and an indication of which observations are assigned to training and which to testing. In this way, trainNN trains a k-NN model with the training set and imputes the response variables from the predictors of the testing set. The function returns a yai object, which is a trained k-NN model, and the predicted and observed forest attributes values of the testing set which can then be compared to assess the model accuracy (see section 2.1.5). Note that when random forest is used to derive the proximity measure, the number of trees and the number of predictor variables randomly selected at each node can be adjusted by the user. Once a k-NN model has been trained on the reference observations and its expected accuracy assessed, it can be used to impute response variables at targets using the predictTrgs function. Given a raster object, where each band is a predictor used to train the k-NN model, the function runs each target cell through the train random forest model and finds its k-nearest reference observations based on the proportion of trees where a reference observation and a target observation fall into the same final node [38]. Once the k-nearest reference observations of each cell has been determined, predictTrgs returns the mapped imputed variables along with the ID of their corresponding nearest reference. Computing the distance between each target cell and the reference observations can require a lot of available memory. By default when using random forest, predictTrgs processes data in chunks to avoid creating big objects (proportional to the number of cells and number of trees) that couldn’t be stored in memory. On small datasets and to speed up calculations, data can be processed on larger chunks but it is advised to keep track of the memory usage. The function tile implemented in FOSTER can also be used to create smaller subsets of data and therefore limit risks of running into memory issues. Parallel processing can also easily be performed with predictTrgs.

#### 2.1.5 Model accuracy assessment

FOSTER provides functions to assess the k-NN model performance using traditional measures of prediction accuracy and scatter plots, as well as evaluate the importance of each predictor in the imputation approach when random forest is used. Given observed and predicted values, the function accuracy computes the coefficient of determination (R^2^), root mean square error (RMSE) and bias, defined as follows:

$$R^{2}=1-\frac{SSR}{SST}=1-\frac{\sum_{i=1}^{n}{\left(y_{i}-{\hat{y}}_{i}\right)}^{2}}{\sum_{i=1}^{n}{\left(y_{i}-{\bar{y}}_{i}\right)}^{2}}$$ (1)

$$RMSE=\sqrt{\frac{1}{n}\sum_{i=1}^{n}{\left(\hat{y_{i}}-y_{i}\right)}^{2}}$$ (2)

$$bias=\frac{1}{n}\sum_{i=1}^{n}\left(\hat{y_{i}}-y_{i}\right)$$ (3)

where *SSR* is the sum of squared residuals, *SST* the total sum of squares, *y_i_* the observed response variable and ${\hat{y}}_{i}$ the imputed value (prediction of the k-NN imputation model). Both relative RMSE and bias are also calculated relative to the mean of the observed values. The accuracy assessment can be performed either on a single held-out subset of the observations or using a k-fold cross-validation approach. The function partition, a wrapper of functions from caret package [45] can be used to split the observations into training and testing sets. To visualize the model performance, the function scatter creates a scatterplot between predicted and observed values and can display a set of accuracy metrics on the plot.

If using random forest proximity matrix as a measure of nearness, each predictor importance in the imputation model can be returned and plotted using varImp function. In a random forest regression or classification, the importance of each predictor is assessed by calculating how much the Out-Of-Bag (OOB) prediction accuracy (defined as the error rate for classification and mean square error for regression) decreases when this predictor is permuted while others are unchanged [43]. The importance value of each predictor variables can be standardized using the z-score (centered by the mean and scaled by the standard deviation of all the predictor importance values). The standardized importance values therefore represent the distance between the importance of a predictor and the average importance of all predictors combined, in terms of standard deviation. The scaled importance is negative when the predictor is below the mean importance and positive when above.

### 2.2 Example of ALS metrics imputation using FOSTER

The following will illustrate how FOSTER can be used, from data preprocessing to imputation, using a test site located in British Columbia, Canada. The objective of this example is to impute two ALS-derived metrics using a 25-year time series of spectral indices and topographic variables as predictors. FOSTER includes a vignette that goes through the example presented herein in more details. All the scripts used to run this example are included in the vignette and the data is included in the package.

#### 2.2.1 Input data

**Fig 2:**
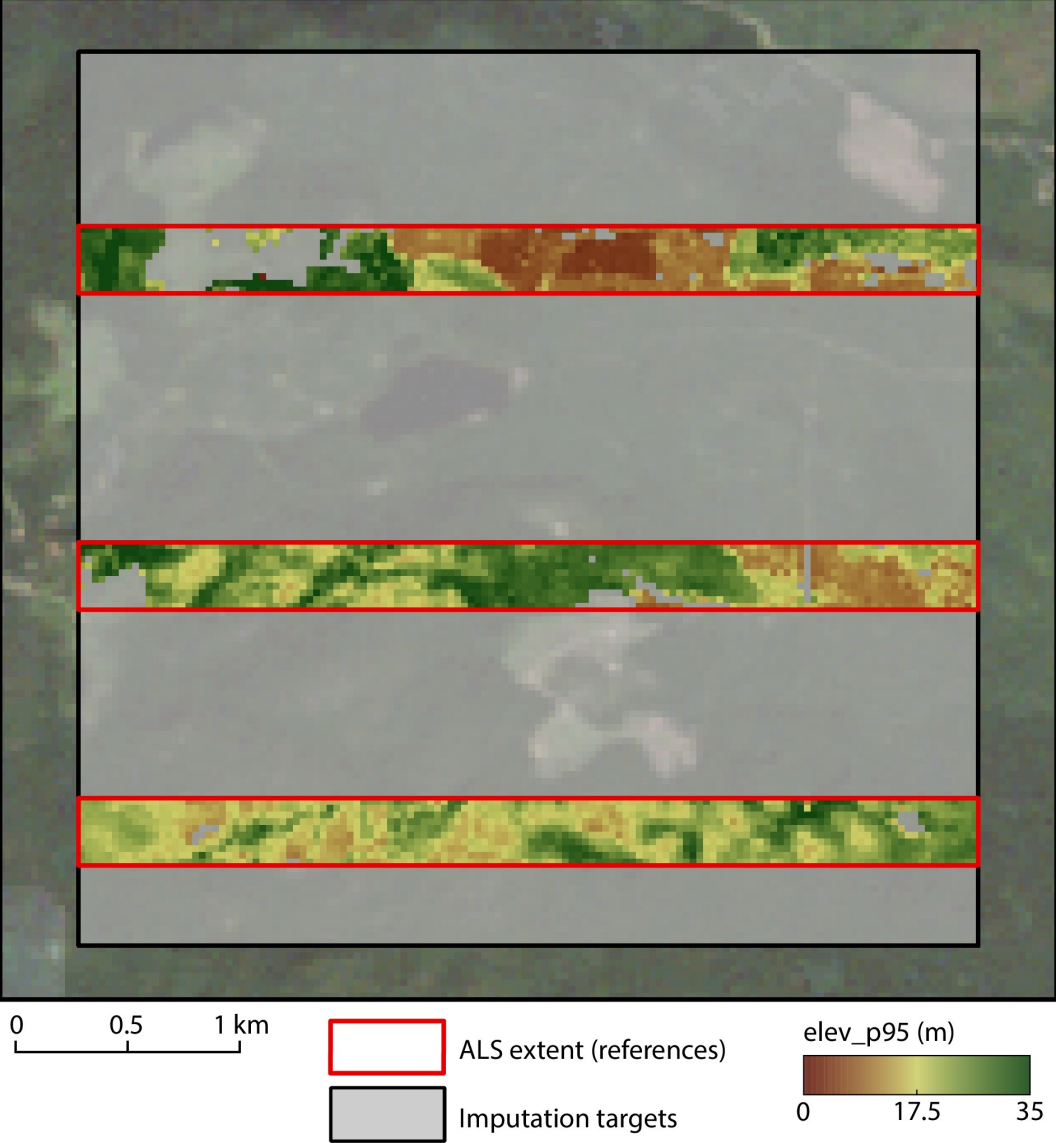
Study area in Alex Fraser Research Forest (British Columbia, Canada). Blocks in red represent the ALS coverage and the light grey box is the area where ALS metrics need to be extrapolated. Predictor variables are available across the entire study area, at both references and targets.

*ALS metrics*. ALS data was acquired in 2008 in the Alex Fraser Research Forest (AFRF) in Canada, BC. The acquisition covered an area of approximately 12 km x 6 km, but this study is focused on a 4 km x 4 km subset (see Fig 2). The 95^th^ percentile of first return heights (*elev_p95*) and the canopy cover above mean height (*cover*) were calculated on a 20 m x 20 m grid using the lidR package [26]. Forest inventory plots in Canada are generally measured using a fixed radius of 11.28 m, corresponding to an area of 400 m^2^ [46]. When generating maps of forest attributes following an area-based approach, it is crucial that the resolution of the grid corresponds to the inventory plots area as closely as possible [6]. Hence 20 m x 20 m gridded lidar metrics are generally used. Three stripes of 500 m width and 4 km length were extracted from the wall-to-wall metrics to simulate discontinuous acquisition blocks. The target cells for imputation are the cells of the 4 km x 4 km area that are not covered by the ALS stripes. The wall-to-wall ALS metrics will only be used after imputation has been performed to assess its performance. Description and summary statistics of *elev_p95* and *cover* are provided in Table 2.

**Table 2:** Summary statistics of the two response variables *elev_p95* and *cover* at the ALS stripes.

Variable	Variable name	Description	Min	Mean	Max	Standard deviation
95^th^ percentile of canopy height	*elev_p95*	95^th^ percentile of returns height	0.3 m	19.9 m	43.9 m	8.5 m
Canopy cover above mean height	*cover*	Proportion of first returns above mean returns height	5.0%	61.2%	91.1%	14.9%

*Landsat time series*. Landsat time series predictors with a resolution of 30 m x 30 m were derived from 25 yearly Landsat composite images for 1984 to 2008 produced following the Composite 2 Change (C2C) approach [47,48]. Specifically, best-available pixel (BAP) image composites were first produced from Landsat imagery by selecting observations for each pixel within a specific date range (August 1+/-30 days) based on the scoring functions defined by [49], which rank the presence and distance to clouds and their shadows, the atmospheric quality, and the acquisition sensor. Next, these image composites were further refined by removing noisy observations (e.g., haze and smoke) and infilling data gaps using spectral trend analysis of pixel time series [35]. This process is not part of FOSTER hence we expect annual time series of optical imagery to be available.

*Topographic data*. The Advanced Spaceborne Thermal Emission and Reflection Radiometer (ASTER) global Digital Elevation Model (GDEM, v.2), with a spatial resolution of 30 m and aligned on Landsat grid was used to derive terrain elevation and slope (in degrees). This standard global product is generated using stereo-pairs of images collected by ASTER on Terra satellite [50].

#### 2.2.2 Description of the main processing steps

The imputation of ALS metrics was carried out following the steps of the diagram shown in Fig 1. Using matchResolution and matchExtent, the ALS metrics were resampled and cropped to the spatial resolution and extent of the 30 m x 30 m Landsat grid. A land cover dataset produced by classifying the annual Landsat BAP images [51] was used to restrict the analysis to forested cells (classified as coniferous, broadleaf or mixedwood). Tasseled Cap Brightness (TCB), Greenness (TCG), Wetness (TCW), which are calculated from a transformation of Landsat spectral bands feature space [52,53] and NDVI were calculated on forested cells for each Landsat image from 1984 to 2008 (ALS acquisition year) using calcIndices as shown below:

# Open all Landsat images in a list (path_BAP contains the path to each Landsat BAP image in chronological order)

BAP_ts <- lapply(path_BAP, raster::stack)

# Calculate spectral indices

calcIndices(BAP_ts, indices = c("NDVI", "TCB", "TCG", "TCW"), = 3, nir = 4, sat = "Landsat5TM")

As suggested by [28] and [23], datasets were smoothed with focalMultiBand using a 3x3 window, assigning a 90 x 90 m average to each cell in order to improve model accuracy. After removing the cells adjacent to forested area borders with edges, the function getSample was used to stratify the two ALS metric maps into 5 clusters and to select 230 cells located at least 75 m from each others (see code below). Due to the small extent of the study area, only a relatively small number of closely spaced cells could be selected. However, it is advised to increase the sample size and the minimum distance between sample points to minimize spatial autocorrelation.

# Stratified random sampling

sample_strata <- getSample(ALS_metrics, layers = c("p95","cover"),n = 230, strata = 5, mindist = 75)

The median, interquartile range (IQR) and Theil-Sen slope of each spectral index time series was calculated at both sample and target locations using temporalMetrics as shown below:

# Example of NDVI time series (NDVI_ts) temporal summary metrics calculation

temporalMetrics(NDVI_ts, metrics = "defaultTemporalSummary")

A total of 14 predictor variables were gathered: the median, IQR and Theil-Sen slope of the 4 spectral indices, the DEM and the terrain slope (summarized in Table 3). The function partition with the "kfold" method was used to split the 230 sampled observations into 5 training sets (80% of observations for each folds, n = 184) and 5 testing sets (remaining 20% of observations for each fold, n = 46). The partition is performed in a manner that maintains a balanced distribution of the k-means clusters, used for stratified random sampling, within each fold. That way, the set used for validation is representative of the entire range of forest structure of the sample and the accuracy assessment is representative of how the model would perform for all types of forest structure in the area. The following code illustrate how the partition function is used:

# Partition sample_strata into 5 folds (sample_strata$cluster contains the strata of each sampled point)

train_folds <- partition(sample_strata$cluster, type = "kfold", kfold = 5)

**Table 3:** Description of the 14 predictor variables used in the k-NN model.

Variable	Variable name	Units	Description
Temporal metrics of TCB, TCW and TCG	*TCB_median(slope)(IQR)*	-	Median, Theil-Sen slope and IQR of 25 year time series of Tasseled Cap indices and NDVI.
	*TCW_median(slope)(IQR)*		
	*TCG_median(slope)(IQR)*		
	*NDVI_ median(slope)(IQR)*		
Elevation	*DEM*	m	Terrain elevation above sea level
Slope	*DEM_slope*	°	Topographic slope in degrees

Using trainNN, a k-NN model was trained from the 14 predictor variables and 2 response variables extracted at training sample points, setting the number of neighbors to k = 1. The random forest proximity matrix was used as the distance measure with the number of trees drawn for each response variable set to 200 and the number of predictors randomly selected at each split set to 3 (default value from random forest).

# Train a kNN model. X_vars_sample and Y_vars_sample are the predictor variables (X) and response variables (Y) extracted at sampled points

kNN_model <- trainNN(x = X_vars_sample, y = Y_vars_sample, k = 1, inTrain = train_folds, ntree = 200)

Finally, the function predictTrgs was used to impute simultaneously *elev_p95* and *cover* and return their mapped predicted values at targets.

# Impute response variables. X_vars is a RasterStack containing all predictor variables

Y_imputed <- predictTrgs(model = kNN_model$model, x = X_vars)

### 2.3 Computing times measurements

The computing time for raster processing functions vary depending on the size of the inputs (e.g. size of the study area), the number of outputs to process (i.e. number of indices, length of time series) and the parameters used for calculation (e.g. type of temporal summary metrics, number of threads in parallel processing). Functions that are likely to take the longer time to run are calcIndices, temporalMetrics and predictTrgs. In order to assess the performance of FOSTER under different conditions, we extracted 5 Landsat BAP images with dimensions of 500 x 500, 1000 x 1000, 2000 x 2000, 3000 x 3000 and 4000 x 4000 pixels (equivalent to 225 km^2^, 900 km^2^, 3600 km^2^, 8100 km^2^ and 14400 km^2^ respectively) and calculated TCG, TCW, TCB, NDVI at 25 different years. In addition, temporal summary metrics spanning the 25 years were generated for each of the four spectral metrics. The k-NN model trained for the example of the Alex Fraser Research Forest was also used to impute response variables on each dataset. The built in capacity of the raster package to perform parallel processing, which has been adapted to FOSTER, was used to assess its influence on computing time. Processing of each dataset was performed with a single thread, 5 parallel threads and 10 parallel threads. Note that the number of rows processed at a time when using predictTrgs was adjusted so that approximately the same number of cells are processed at the same time for each dataset (50, 25, 12, 8 and 6 rows from the smallest to largest dataset). Computing times required to process the data and write the output to file were tracked using the R package microbenchmark [54]. Computations were performed using a desktop PC with Windows 10 Enterprise 64-bit, a dual Intel® Xeon® E5-2630 0 @ 2.30GHz CPU (12 cores, 24 logical processors) and 64.0 GB RAM DDR3.

## 3 Results

### 3.1 Computing time measurements

**Fig 3:**
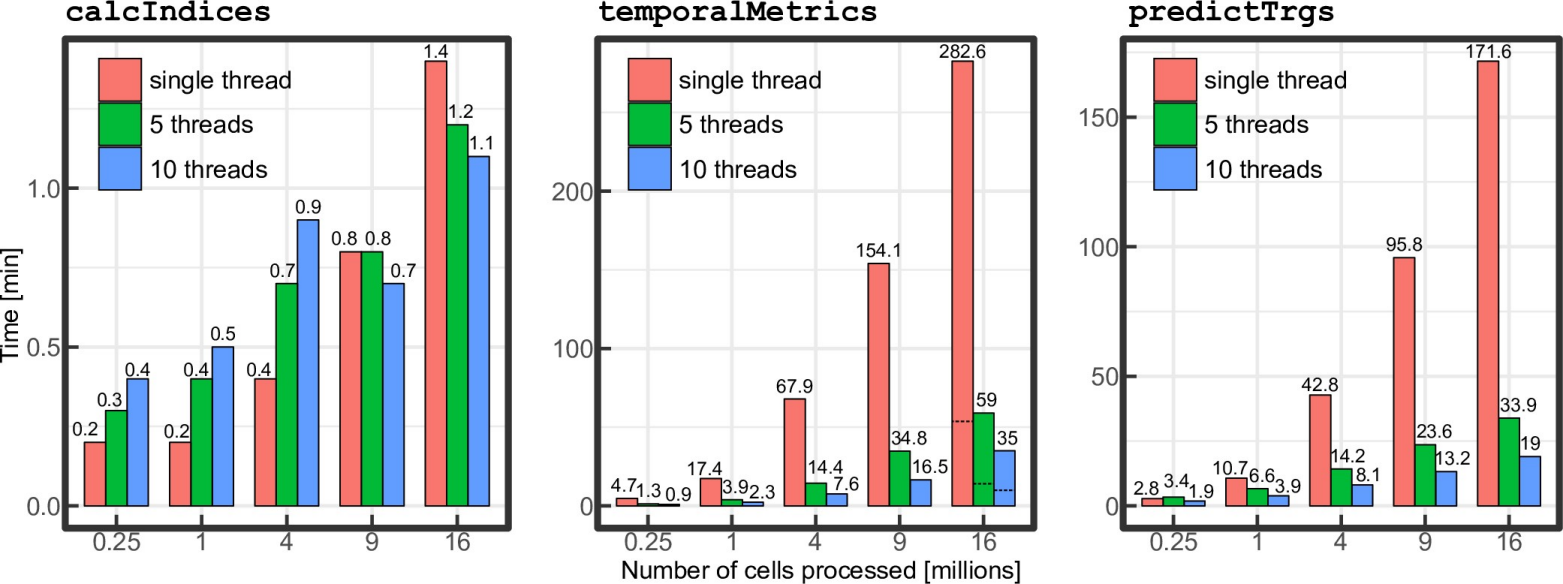
Computing times, in minutes, required to process calcIndices, temporalMetrics and predictTrgs with 5 datasets of different sizes on single and multiples threads. Dashed lines for the 4000 x 4000 pixels in temporalMetrics represent computing times without calculating the Theil–Sen slope.

Computing times measured for the function calcIndices, temporalMetrics and predictTrgs are reported in Fig 3. Calculating TCG, TCB, TCW and NDVI with calcIndices is the fastest process with computing times ranging from 10.3 s to 81.8 s on a single thread. Computing times for parallel execution of calcIndices increase for the 500 x 500, 1000 x 1000 and 2000 x 2000 datasets and start to slightly decrease for the 3000 x 3000 and 4000 x 4000 datasets. Advantages of parallelization are much more important for the two other functions temporalMetrics and predictTrgs. On 5 and 10 parallel threads, computing times of both functions are reduced by around 80% and 90% respectively when processing the 3000 x 3000 and 4000 x 4000 datasets. Computing times for temporalMetrics ranges from 4.7 min– 282.6 min on a single thread, 1.3 min– 59 min on 5 threads and 0.9 min– 35 min on 10 threads. The type of temporal summary metrics calculated can also have a large influence on the computing times. For example, calculating only the median and IQR reduces computing times by around 80% on the larger dataset compared to calculating median, IQR and Theil-Sen slope. Compared to single thread processing, using 5 and 10 parallel processing threads resulted in mean processing time reduction (calculated only when using parallel processing is faster than single thread) of 14% and 17% for calcIndices, 77% and 87% for temporalMetrics and 65% and 70% for predictTrgs.

### 3.2 Imputation example

**Fig 4:**
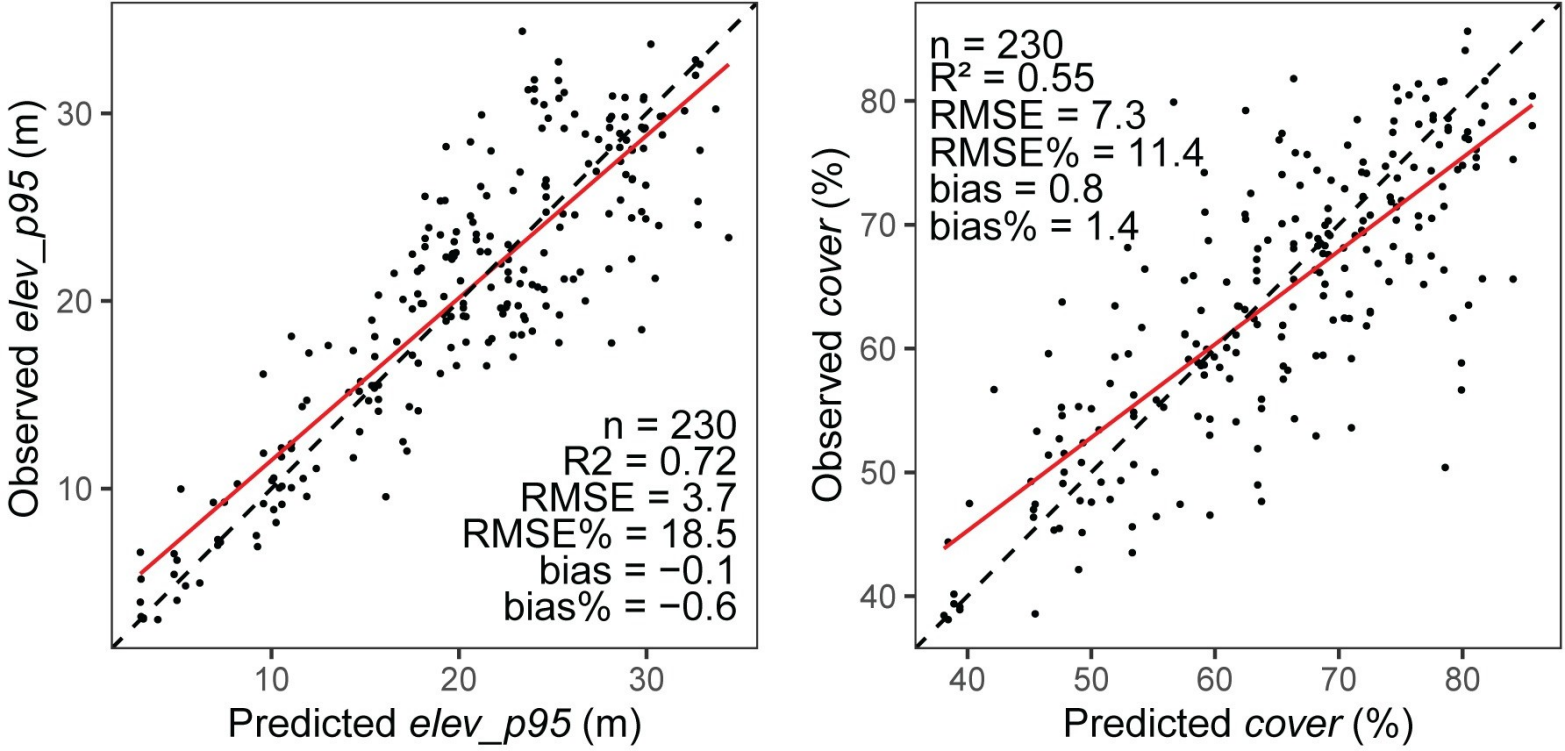
**Scatterplots between observed and predicted *elev_p95* (left) and *cover* (right).** The 5 folds of the cross-validation are included in the plots. The solid red lines indicate the best linear regression fit.

Averaged over the 5 cross-validation folds, the model predicts *elev_p95* and *cover* with a R^2^ value of 0.72 and 0.55, RMSE% of 18.5% and 11.4% and a relative bias of -0.6% and 1.4% respectively. Scatterplots between predicted and observed ALS metrics for the 5 folds are displayed in Fig 4. A R^2^ value of 0.60 and 0.47, RMSE% of 26.1% and 12.6% and a relative bias of 3.2% and 1.5% were obtained when comparing predicted and observed *elev_p95* and *cover* over the entire imputed area (see S1 Fig for corresponding scatterplots).

**Fig 5:**
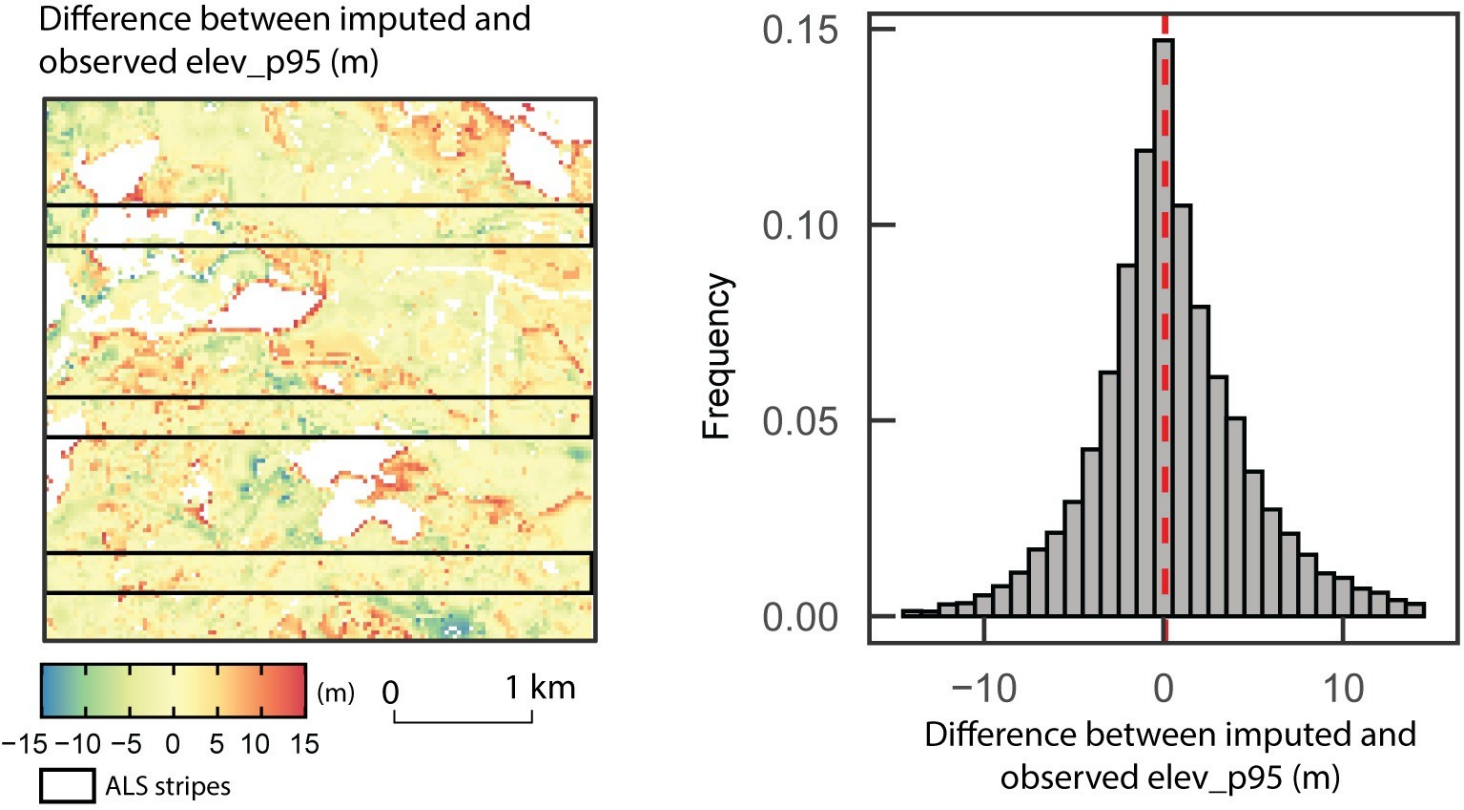
**Mapped difference between predicted and observed *elev_p95* values (left) and associated residuals distribution (right).** Observed *elev_p95* is extracted directly from wall-to-wall ALS acquisition across the study area.

**Fig 6:**
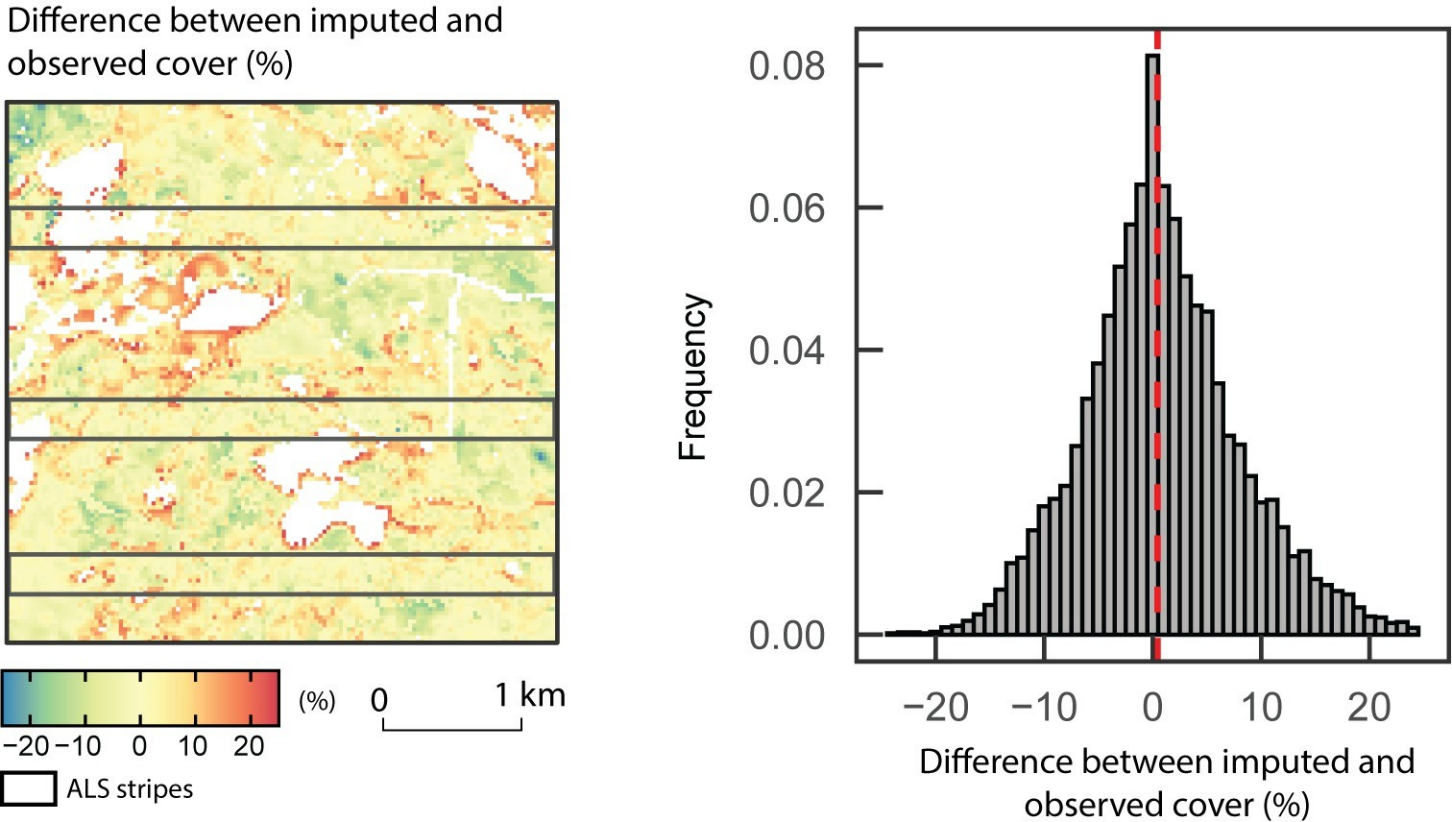
**Mapped difference between predicted and observed *cover* values (left) and associated residuals distribution (right).** Observed *cover* is extracted directly from wall-to-wall ALS acquisition across the study area.

The mapped differences between predicted and observed *elev_p95* and *cover* values with their associated residuals distribution are shown in Figs 5 and 6. The output of predictTrgs giving the nearest reference neighbour ID of each cell indicates that the response variable values of the 230 reference observations used to train the k-NN model were all imputed at least once. Global patterns are relatively well captured by the imputation model, with a gradient in forest structure from short stands in the northeast to taller stands in the west and south. Larger differences between observed and predicted attributes occur mostly at the boundary of forested stands with either water or bare land. Some important differences also occur in the southwest of the area, where patches of high and low canopy height are intermingled. Over the entire imputation target, the mean and maximum absolute difference between the imputed and ALS-derived metrics are 3.3 m and 29.5 m for *elev_p95* and 5.6% and 49.7% for *cover* respectively.

**Fig 7:**
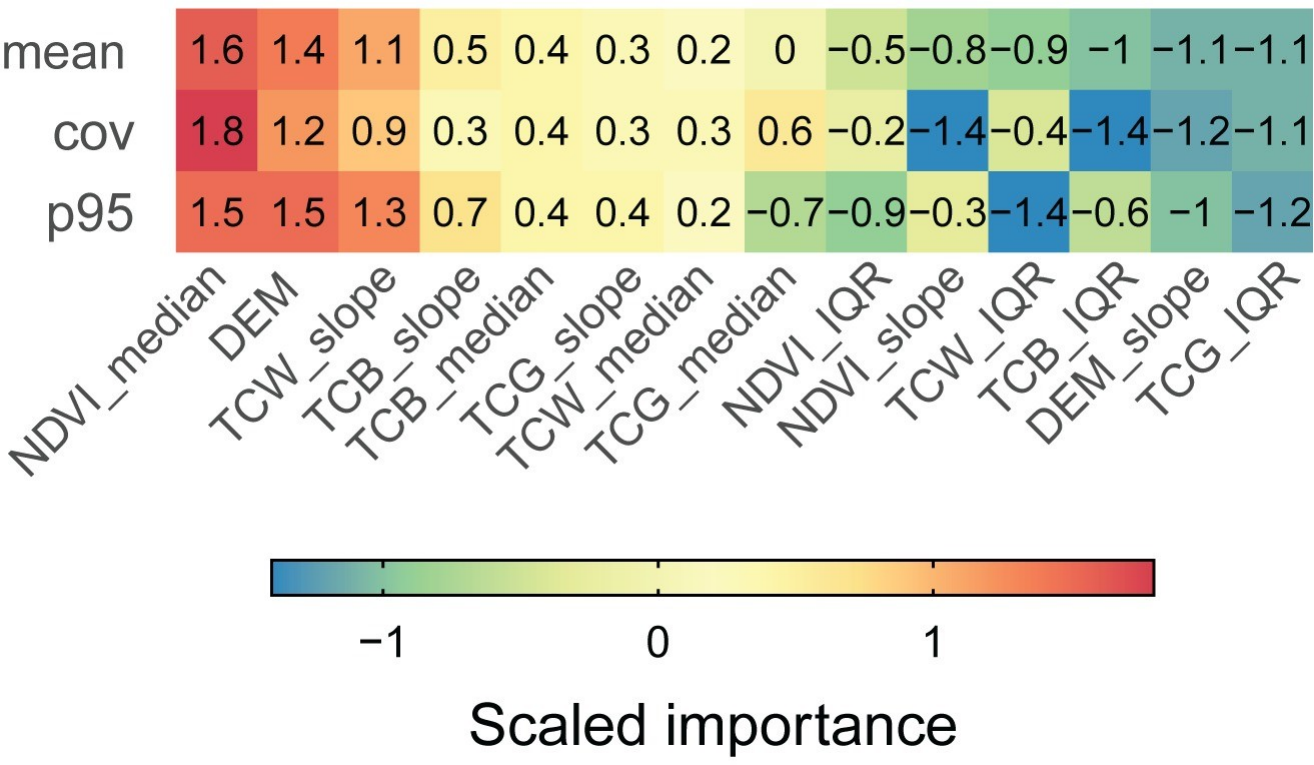
Scaled importance and mean scaled importance of the predictors for each response variable.

As shown in Fig 7, the most important variable for the random forest predictions of *cover* and *elev_p95* is the 25 year median of NDVI. The Theil-Sen slope and median of the 25 year spectral indices time series are generally more important than IQR metrics which all have a negative scaled importance. The terrain elevation (DEM) is the second most important variable for both *cover* and *elev_p95* but terrain slope (DEM_slope) has little importance in this example.

## 4 Discussion

The framework implemented in FOSTER has been designed for the imputation of 3D forest attributes using predictor variables derived from annual multispectral time series and ancillary data within a single computing environment, R. The processing workflow presented herein intends to provide a general method that researchers wanting to extrapolate ALS or DAP derived data over large areas using k-NN imputation can follow.

While the purpose of the example presented in this paper is to illustrate how to use FOSTER rather than optimizing the model, both predicted response variables achieved relatively good accuracy. However, larger imputation problems might suffer from different issues. First, since imputation models do not extrapolate beyond the reference observations range, it is important to ensure that they capture the entire structural variability range expected across the target region. Similarly, the stratified sample should be selected with care to produce meaningful results. Spatial autocorrelation within the sample can be minimized by introducing a minimum distance between sample location points. However, in an environment where forest structure is highly clustered this might not be sufficient. In the example presented herein, the minimum distance between sampled points was limited by the small extent of the simulated ALS stripes. It is therefore likely that effects of spatial autocorrelation were not removed and thus lead to overoptimistic accuracy metrics. Then, the topography and disturbance regime of the study area will drive the choice of predictor variables and the optimal time series length. For example, terrain elevation is often an important variable to include to characterize forest structure variability [23,24,32]. Intensively managed stands might also benefit from information on disturbance history by including variables like years since last disturbance or disturbance type. Knowledge of the area would generally inform on the most relevant types of spectral indices to use.

The extrapolation of forest attributes with Landsat derived predictors and k-NN imputation is one of the many possible approaches. For example, Chi et al. [55] and Sun et al. [56] extrapolated lidar-derived estimates of above ground biomass from MODIS and SAR respectively using random forest regression. Although only k-NN imputation has been presented here and is currently fully implemented, FOSTER provides the flexibility to integrate any type of remote sensing data and choose other extrapolation methods than k-NN that are integrated in the yaImpute package. As pointed out by Eskelson et al. [57], advantages of k-NN methods are that they are non-parametric, multivariate and are not limited by assumptions regarding the distribution of the response variables. When using *k = 1*, k-NN models ensure that the natural variation and allometric relationships (for example basal area to height ratio) among forest attributes is retained. Using k *> 1* results in smoothing, hence producing estimates with lower variance however illogical relationships between estimated response variables might occur [57]. The choice of nearness measure also influences the accuracy of the estimates. Hudak et al. [39] showed that the use of the random forest proximity matrix as a nearness measure outperformed methods using other types of measures like euclidean or mahalanobis distance.

In FOSTER, the focus is on the imputation rather than estimation of forest attributes from ALS or DAP derived data. However, the confidence in the extrapolated forest attribute maps highly is dependent on the accuracy of the inputs. In two-stage modeling approaches, where response variables are modeled, the variance in the final prediction is underestimated if the modeling uncertainty is not considered. Saarela et al. [58] proposed a new estimator accounting for the uncertainty of the intermediate model when ordinary least square regression models are developed for both modeling stages. However, such an estimator for two-phase modeling with non-parametric approaches has not yet been derived. We therefore emphasize on the fact that accuracy metrics reported by FOSTER only account for the k-NN imputation model and might not reflect the accuracy of the final extrapolated forest attribute maps. Additional field-based validation data within the extrapolated area would be necessary to assess the accuracy of the modeled response variables beyond the modeling process.

Computing time measurements reported in this paper show that imputation can be performed in a reasonable amount of time. Memory issues are well handled by the raster package on which the FOSTER package relies on for reading, processing and writing raster data. The most sensitive step concerning memory issues is when imputing response variables with predictTrgs. Choosing the right tile size and optimal number of rows that can be processed at the same time depends on the memory available on the machine used.

The framework currently implemented in FOSTER is one of the many possible ways to extrapolate forest structural attributes with wall-to-wall satellite data. We recognize that modifications to the workflow presented herein, such as the integration of highly detailed structural attributes with coarser satellite data and other sampling approaches, such as Halton sequences instead of random sampling, may be preferred by some users. The open source nature of the code allows other approaches to be integrated into the workflow. With respect to further FOSTER development, additional functionalities and methods could be implemented in the future based on users needs and feedback.

## 5 Conclusion

Despite the increasing collection rate of ALS and DAP for forest inventory purposes, covering large areas remains expensive. In order to take advantage of this highly detailed data, imputation can be used to estimate 3D forest attributes on a large scale by making use of wall-to-wall satellite imagery acquired regularly over the past decades. FOSTER is innovative thanks to its implementation of a robust sampling approach, the integration of computationally efficient calculation of spectral trends summary metrics and its state of the art implementation of a full framework where k-NN imputation can be performed.

To illustrate how to use FOSTER, an example of the imputation of ALS derived metrics using a 25-year Landsat time series and topographic variables was performed using only functions implemented in the package. Computing time measured on application datasets show that the entire process can be performed in a reasonable amount of time and that the simple parallel processing implementation can greatly increase performances. We hope that FOSTER will be useful to researchers and that further developments will adjust the number of functionalities and computation efficiency to suit users’ needs.

## Supporting information

S1 Fig (TIF)
