# Supplementary figures and images for: FOSTER—An R package for forest structure extrapolation
Source: PLoS One. 2021 Jan 28;16(1):e0244846. doi: 10.1371/journal.pone.0244846 (PMC7842971; doi:10.1371/journal.pone.0244846)

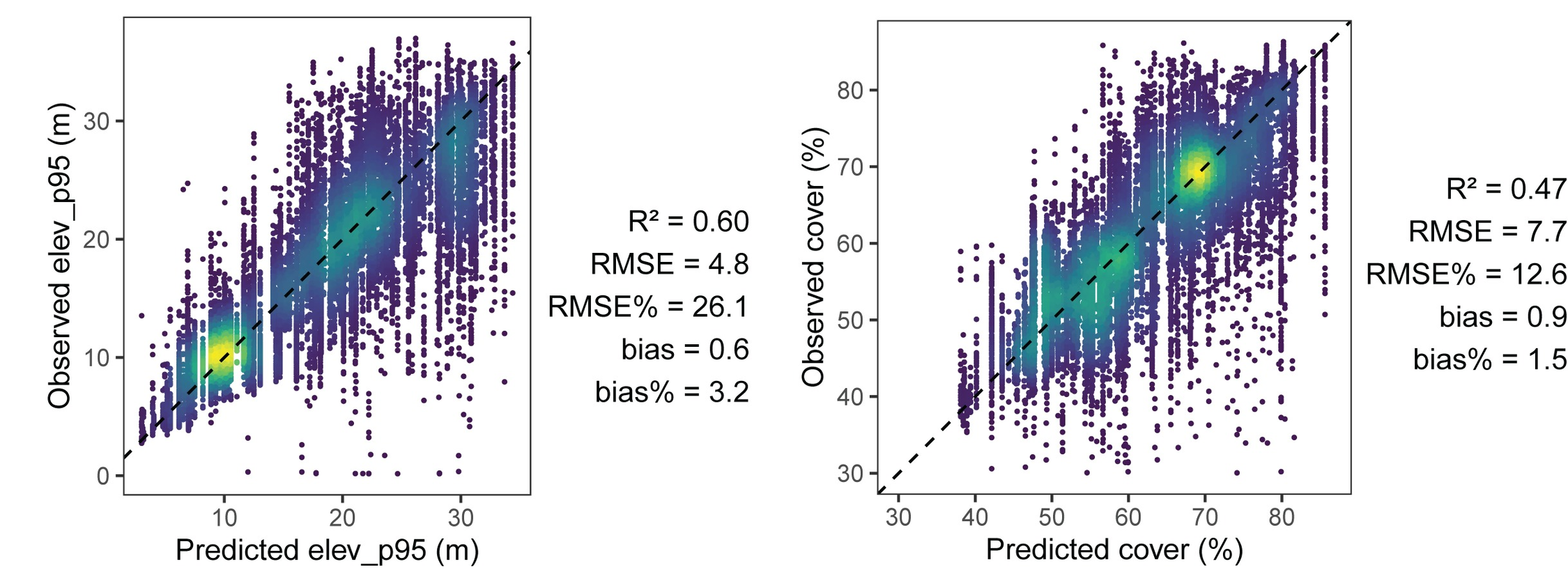

Supplement: S1 Fig — (TIF) [file pone.0244846.s001.tif]
